# A scoping review on research agendas to enhance prevention of epidemics and pandemics in Africa

**DOI:** 10.11604/pamj.supp.2020.37.1.23458

**Published:** 2020-11-24

**Authors:** Jill Ryan, Alison Wiyeh, Humphrey Karamagi, Joseph Okeibunor, Prosper Tumusiime, Charles Shey Wiysonge

**Affiliations:** 1 Cochrane South Africa, South African Medical Research Council, Cape Town, South Africa,; 2 Department of Epidemiology, University of Washington, Seattle, Washington, USA,; 3 World Health Organization, Regional Office for Africa, Brazzaville, Congo,; 4 School of Public Health and Family Medicine, University of Cape Town, Cape Town, South Africa,; 5 Department of Global Health, Stellenbosch University, Cape Town, South Africa

**Keywords:** Research agenda, pandemic, epidemic, prevention, Africa

## Abstract

**Introduction:**

research is not only needed to prioritise the best possible response during an epidemic and pandemic, it is also understood to be a core pillar of outbreak response. However, few African countries are equipped to perform the needed surveillance and research activities during an outbreak. Therefore, we mapped out research agendas aimed at increased research preparedness towards epidemics or pandemics in Africa.

**Methods:**

eligible studies were searched for in in PubMed, Scopus, and Google Scholar. Additionally, grey literature was sought in Google, citation searches, as well as targeted sites such as the World Health Organization (WHO), Africa Centres for Disease Control and Prevention, African Union, and the Wellcome Trust. Searches were done in March 2020.

**Results:**

the electronic searches yielded 7344 records, of which 34 articles were included in the study. The studies identified around 18 factors highlighted through various research agendas. Majority of the research agendas spoke to general epidemic preparedness and focused largely on understanding virus transmission such as its characteristics and dynamics, and the infrastructure needed to carry out vital research activities.

**Conclusion:**

the review highlights the research needs in order to carry out vital research work but to also bridge knowledge gaps and harmonize outbreak response from key stakeholders. However, Africa needs to create its own health research agendas and capacitate itself to conduct and lead these studies. African health research decisions must center on Africa, with African researchers taking the lead not only on the science produced but ensuring inclusive and equitable involvement from fellow researchers, and in engaging national health ministries as well as the communities.

## Introduction

Conducting valuable research during pandemics and epidemics is not only needed to prioritize the best possible response but is also acknowledged as a core pillar of outbreak response [1]. During recent public health crises, such as the 2009 H1N1 influenza pandemic, government leaders needed to make decisions at a time where information was limited and highly uncertain [2]. Effective processes were needed between stakeholders to facilitate the utilization of scientific information to assess each scenario, and ultimately identify interventions offering the most beneficial outcomes [2]. However, with emerging and re-emerging infections such as Ebola, Zika, Influenza, and the subsets of coronavirus, there are challenges regarding rapid research output or to rapidly undertake intervention trials to inform practice and patient care [1,3]. These challenges are linked to the unpredictable nature of the virus, as well as its time and location of onset [1,3]. Thus, the need for scientific and research preparedness is unquestionable in its necessity when prioritizing effective outbreak responses [1,2].

The World Health Organization (WHO) has indicated its awareness regarding the need for such preparedness, and in a 2018 document on managing epidemics, they mention their global strategy and preparedness plan highlighted as their research and development blueprint [4]. The strategies highlighted in this document will aim to allow rapid activation of research and development activities which will fast-track the availability of effective diagnostic tests and treatment, averting large scale crises [4,5]. However, when epidemics overlap and occur at the same time, the challenge is creating an efficient global response to increase multiple public health emergencies. Such a response requires integration from health, economic and global systems to prepare for said epidemics or outbreaks [6]. This proves difficult when not all countries are equipped to carry out these research activities and required surveillance, as is the case in many low- and middle-income settings in the African continent.

Outbreaks in Africa have shown time and time again, to be a cause of global concern [7]. Out of the 55 African countries, only 15 currently have institutions equipped to perform the functions of an effective National Public Health Institution (NPHI) which includes disease surveillance of diagnostic laboratories, capacity to activate response teams for outbreaks, as well as being operation centres for public health crises [7]. Equipping African NPHIs is vital for health security [7,8]. With Africa´s 1.2 billion population expected to expand to 2.4 billion by 2050, this rapidly expanding population means greater urbanization but also increased informal settlements; where, already half of urban areas do not have access to sanitation, and even less have access to scheduled childhood vaccinations [7]. Furthermore, rapid population increase, and urbanization also means increased risk for outbreaks and limited response to address it. This is due primarily to Africa´s incapacity to swiftly and effectively manage these emergencies [7,8]. The Africa Centres for Disease Control and Prevention (Africa CDC) estimates that Africa requires 2-3.5 billion US dollars per year to be adequately capitated for epidemic preparedness, yet only receives $700 million [7]. In this scoping review, research agendas aimed at increasing research preparedness toward epidemics or pandemics, which may facilitate and streamline effective response for emerging and re-emerging disease outbreak were mapped.

## Methods

A scoping review maps the existing literature on a given topic and identifies key concepts and gaps in the existing research on that topic. In this scoping review we followed the methods proposed by Levac *et al*. [9] and enhanced by Peters *et al*. [10]. We have used the preferred reporting items for systematic reviews and meta-analysis-extension for Scoping reviews (PRISMA-ScR) checklist to shape structure and content [11].

**Eligibility criteria:** the inclusion criteria are structured according to the components suggested in Peters *et al*. [10]. Inclusion criteria were broken down into the following elements: participants, concepts, and context.

**Participants:** African “think tank” organizations: African Union, WHO, United Nations (UN), United Nations Children Fund (UNICEF), and universities, focusing research on Africa.

### Concepts

*Research agendas:* research strategies which facilitate preparedness.

*Epidemic:* “a disease (infectious and noninfectious) that affects a large number of people, with a recent and substantial increase in the number of cases” [12].

*Pandemic:* an outbreak with characteristics which include wide geographic expansion, disease movement which can be traced from place to place, has high attack rates and explosiveness (multiple cases within short space of time); minimal population immunity; novelty (new or new variants of pathogen); it is infectious as well as severe [13].

**Context:** within Africa; no specificity related to cultural factors, racial or sex-based factor, setting such as a health facility or school or discipline.

**Search strategy:** one author (JR) conducted a search of PubMed, Scopus, and Google Scholar in March 2020. She also searched grey literature such as technical reports, research reports, committee meeting reports, conference proceedings, abstracts, and dissertations on websites identified to relate to research agendas and outbreaks in Africa. Grey literature from relevant organization sites were hand searched and snowballing technique used when going through relevant documents found. Grey literature sites included WHO, Africa CDC, African Union, and the Wellcome Trust. Literature published within a 10-year time span from date of this current review (2010 - 2020) to elicit latest trends addressing recent response outbreak needs. Keywords used for index search were ''research'', ''outbreak preparedness'', ''research priorities'', ''epidemic response'', ''pandemic response'', ''real-time research'', and ''Africa''. Furthermore, the search term ''Africa'' was further expanded to include all African countries through a search strategy filter created by Pienaar and colleagues [14]. The search strategy for PubMed is shown in Table 1. In addition to searching organization websites for relevant documents, not all of the organization documents presented in the website search, and as such documents, for example from WHO, were also searched for through Google.

**Table 1:** search strategy for PubMed

Search string	Limiters
Research preparedness and outbreaks and (((Algeria) or (Angola) or (Benin) or (Botswana) or (Burkina Faso) or (Burundi) or (Cameroon) or ((Canary Islands) or “Canary Islands”) or ((Cape Verde) or “Cape Verde”) or (Central African Republic) or (Chad) or (Comoros) or (Congo) or (Democratic Republic Congo) or (Djibouti) or (Egypt) or ((Equatorial Guinea) or “Equatorial Guinea”) or (Eritrea) or (Ethiopia) or (Gabon)) or ((Gambia) or (Ghana) or (Guinea) or ((Guinea Bissau) or “Guinea Bissau”) or (Ivory Coast) or ((Cote D'ivoire) or “Cote D'ivoire”) or ((Cote Ivoire) or “Cote Ivoire”) or (Kenya) or (Lesotho) or (Liberia) or (Libya) or (Libia) or (Jamahiriya) or (Jamahiryia) or (Madagascar) or (Malawi) or (Mali) or (Mauritania) or (Mauritius) or (Morocco)) or ((Mozambique) or (Mocambique) or (Namibia) or (Niger) or (Nigeria) or (Reunion) or (Rwanda) or ((Sao Tome) or “Sao Tome”) or (Senegal) or (Seychelles) or ((Sierra Leone) or “Sierra Leone”) or (Somalia) or ((South Africa) or “South Africa”) or ((St Helena) or “St Helena”) or (Sudan) or (Swaziland) or Eswatini or (Tanzania) or (Tanganyika) or (Togo) or (Tunisia)) or ((Uganda) or ((Western Sahara) or “Western Sahara”) or (Zaire) or (Zambia) or (Zimbabwe) or (Africa[Mh]) or (South* and Africa*) or (West* and Africa*) or (East* and Africa*) or (North* and Africa*) or (Central* and Africa*) or (Sub Saharan Africa*) or (Subsaharan Africa*) or (Africa*))) not (((Guinea Pig*) or “Guinea Pig*”) or ((Aspergillus Niger) or “Aspergillus Niger”))	10 years
“Research” priorities and epidemic response and (((Algeria) or (Angola) or (Benin) or (Botswana) or (Burkina Faso) or (Burundi) or (Cameroon) or ((Canary Islands) or “Canary Islands”) or ((Cape Verde) or “Cape Verde”) or (Central African Republic) or (Chad) or (Comoros) or (Congo) or (Democratic Republic Congo) or (Djibouti) or (Egypt) or ((Equatorial Guinea) or “Equatorial Guinea”) or (Eritrea) or (Ethiopia) or (Gabon)) or ((Gambia) or (Ghana) or (Guinea) or ((Guinea Bissau) or “Guinea Bissau”) or (Ivory Coast) or ((Cote D'ivoire) or “Cote D'ivoire”) or ((Cote Ivoire) or “Cote Ivoire”) or (Kenya) or (Lesotho) or (Liberia) or (Libya) or (Libia) or (Jamahiriya) or (Jamahiryia) or (Madagascar) or (Malawi) or (Mali) or (Mauritania) or (Mauritius) or (Morocco)) or ((Mozambique) or (Mocambique) or (Namibia) or (Niger) or (Nigeria) or (Reunion) or (Rwanda) or ((Sao Tome) or “Sao Tome”) or (Senegal) or (Seychelles) or ((Sierra Leone) or “Sierra Leone”) or (Somalia) or ((South Africa) or “South Africa”) or ((St Helena) or “St Helena”) or (Sudan) or (Swaziland) or Eswatini or (Tanzania) or (Tanganyika) or (Togo) or (Tunisia)) or ((Uganda) or ((Western Sahara) or “Western Sahara”) or (Zaire) or (Zambia) or (Zimbabwe) or (Africa[Mh]) or (South* And Africa*) or (West* and Africa*) or (East* and Africa*) or (North* and Africa*) or (Central* and Africa*) or (Sub Saharan Africa*) or (Subsaharan Africa*) or (Africa*))) not (((Guinea Pig*) or “Guinea Pig*”) or ((Aspergillus Niger) or “Aspergillus Niger”))	10 years
Real-time research and pandemic response and (((Algeria) or (Angola) or (Benin) or (Botswana) or (Burkina Faso) or (Burundi) or (Cameroon) or ((Canary Islands) or “Canary Islands”) or ((Cape Verde) or “Cape Verde”) or (Central African Republic) or (Chad) or (Comoros) or (Congo) or (Democratic Republic Congo) or (Djibouti) or (Egypt) or ((Equatorial Guinea) or “Equatorial Guinea”) or (Eritrea) or (Ethiopia) or (Gabon)) or ((Gambia) or (Ghana) or (Guinea) or ((Guinea Bissau) or “Guinea Bissau”) or (Ivory Coast) or ((Cote D'ivoire) or “Cote D'ivoire”) or ((Cote Ivoire) or “Cote Ivoire”) or (Kenya) or (Lesotho) or (Liberia) or (Libya) or (Libia) or (Jamahiriya) or (Jamahiryia) or (Madagascar) or (Malawi) or (Mali) or (Mauritania) or (Mauritius) or (Morocco)) or ((Mozambique) or (Mocambique) or (Namibia) or (Niger) or (Nigeria) or (Reunion) or (Rwanda) or ((Sao Tome) or “Sao Tome”) or (Senegal) or (Seychelles) or ((Sierra Leone) or “Sierra Leone”) or (Somalia) or ((South Africa) or “South Africa”) or ((St Helena) or “St Helena”) or (Sudan) or (Swaziland) or Eswatini or (Tanzania) or (Tanganyika) or (Togo) or (Tunisia)) or ((Uganda) or ((Western Sahara) or “Western Sahara”) or (Zaire) or (Zambia) or (Zimbabwe) or (Africa[Mh]) or (South* and Africa*) or (West* and Africa*) or (East* and Africa*) or (North* and Africa*) or (Central* and Africa*) or (Sub Saharan Africa*) or (Subsaharan Africa*) or (Africa*))) not (((Guinea Pig*) or “Guinea Pig*”) or ((Aspergillus Niger) or “Aspergillus Niger”))	10 years

Google was also used to search for additional grey literature, while searching for missing WHO documents. As noted in a paper by Godin *et al*. [15], Google can be overwhelming owing to the immense information available and inconsistent website organization. For this reason Godin and colleagues further stated that it is impossible to screen all retrieved results and as such one must rely on the power of the relevancy ranking within the Google search engines, which is to bring out the most relevant results to the top of the list [15]. The relevancy list generated by Google, is presented in a set number of pages to be screened in advance, ensuring consistency and time management and thus narrowing the search results to specific subject area allowing refined and targeted searching [15]. The first 10 pages of hits were screened (100 results), through title screen and the short text beneath the title, similar to the method used in Godin *et al*. [15]. The review strategy had been shaped using the PRESS (Peer Review of Electronic Search Strategies) 2015 guideline statement, which allowed the discovery of search errors and offers enhancements to the selection of subject headings and text words, resulting in additional studies [16].

**Study selection:** selection criteria was based on the specifics of the research question and later refined with new familiarity on the subject matter through reading the studies. Clarity in decision-making of selection criteria was clarified using the following steps: i) initial team meetings were conducted every week to discuss inclusion and exclusion criteria. ii) More than one authors was used to clarify situations of disagreement to determine final inclusion. iii) Meetings between two authors (JR and CSW) were held at the beginning, midpoint and final stages of the abstract review process discussing challenges and uncertainties related to study selection and to go back and refine the search strategy if needed.

**Figure 1:**
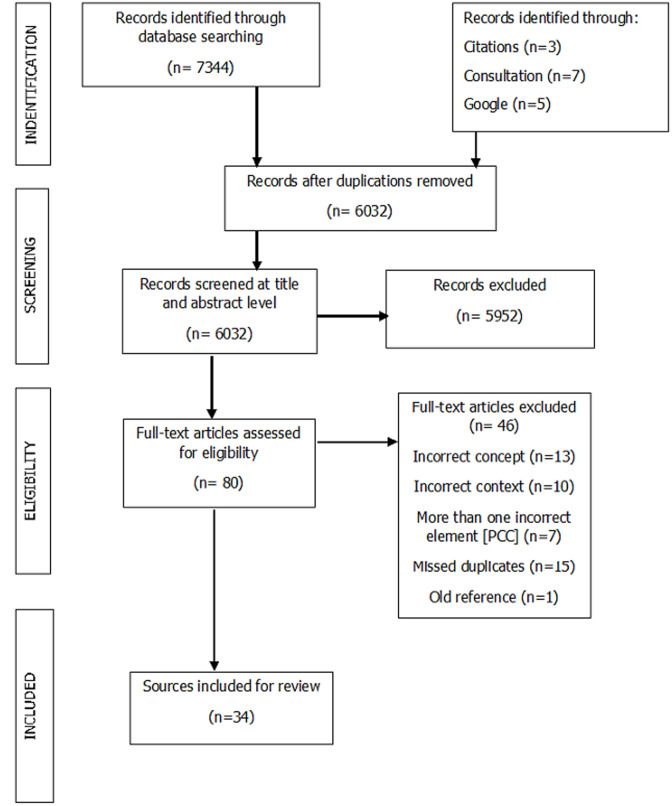
preferred reporting item for systematic reviews and meta-analysis flow diagram

**Extraction and charting the results:** results were extracted and narratively described contextual or process-oriented information from each document, accompanied by a flow chart to show search decision and final selection (Figure 1). Firstly, the data extraction sheet in tabular form summarized each study according to: 1) author(s), 2) year of publication, 3) country of focus, and 9) key message that relate to the review question. Furthermore, the extracted data has been translated into a logical and descriptive summary of the results linked to addressing the review question. As such the results have been presented below through a narrative synthesis.

## Results

**Selection of sources of evidence:** we found a collective 7344 publications from the database search, and an additional 15 documents from other means described in Figure 1. After duplicate removal, 6032 records were screened at title and abstract level, resulting in the exclusion of 5952 records. The remaining 80 articles were assessed for eligibility. Of the 80 titles a further exclusion of 46 articles, with reasons, was undertaken, as shown in Figure 1. One of the reasons for exclusion involved missed duplicates, which can occur as false negatives (duplicates which were to be deleted but were not) in reference managers [17]. After these exclusions, 34 articles were included in the review [18-52].

**Characteristics of sources of evidence:** of the included documents, 22 were journal articles, 7 were research frameworks, 3 were reports, and 2 were commentaries. Majority of the articles assessed the relevance of current epidemic research agendas and its alignment to context and current research needs. Furthermore, the journal article records also aimed in establishing research priorities and in showing the process and tools used to create these priorities. Commentaries focused on having research sites and networks established and strengthened, which would streamline barrier resolutions, case definitions, protocols, and create rapid exchange mechanisms for high quality data and samples to reduce duplication. The frameworks and reports set out establishing research areas for preparedness and in highlighting the need for strengthening infrastructure to carry out the research in a safe and effective manner.

**Synthesized results:**Table 2, Table 3, and Table 4 highlights the key message of each document included in the study. Majority of the studies had focused their research agendas around understanding transmission and infrastructure. Studies which focused on transmission sought to understand the characterization, etiology, or pathogenesis of the disease so as to better identify the pathogen, how it may interplay with other disease (e.g. TB and diabetes or HIV associated TB), as well as to study disease interaction such as vector-virus interaction or if the pandemic goes from region to region, the impact this may have on the pathogen strain [18,30,34,45,48-51]. Infrastructure was seen as vital in many of the documents, which includes creating infrastructure to safely and efficiently carry out real-time research during epidemics, in a way where samples are kept in a safe manner and where cold chain is adequate [26,27,37,40,42-44,47]. Data sharing had been regarded to play an integral role during an epidemic especially to prevent duplication but also to create comparable data sets [21,25,27,36,41,50]. Five sources spoke explicitly on prevention as an important component, but to have contextual consideration when thinking prevention, for example, many forms of cancers which can be prevented, still have high rates in Africa [18,22,33,40,49]. Sensitivity to context had been expressed more in terms of interventions and its implementation, which includes contextualizing well-known or proven interventions which would be closely aligned to population-based information [18,26,46,52]. Treatment had been addressed in diverse ways, either through the inclusion of particular populations (men who have sex with men for appropriate HIV treatment options), to understand co-morbidity (HIV associated TB/TB associated diabetes) for integrated treatment options, to link treatment to understanding transmission, and lastly treatment studies to focus treatment for present-day and not future epidemics, which had been a strong assertion in the Ebola studies as a sentiment from community engagement [22,30,33,39,48].

**Table 2:** summary of evidence for included studies addressing general epidemic research agendas

Author (year)	Region of focus	Type of document	Key message
Zumla (2017)	Africa	Commentary	From a ONE HEALTH partnership “develop and strengthen suitable sites and regional networks to enable resolution of administrative, regulatory, disciplinary, ethical, and cultural barriers; harmonize clinical case definitions and management guidelines; pre-approve adaptable protocols; and introduce mechanisms to rapidly exchange high quality data and samples.” (p607) This in turn would improve the ability to conduct and coordinate large scale studies.
SPRINT-SARI investigators (2018)	sub-Saharan Africa/Global	Journal article	Urges for globally applicable severity of illness score at admission for adults and children as to perform valid international comparative studies of patients.
Moon (2017)	West Africa	Journal article	Finance and technical assistance available for outbreak response; tackle unwarranted trade and travel restrictions; international standards needed for data and information sharing as well as finance for R&D regarding outbreak response beyond vaccines and therapeutics; for WHO to tackle institutional weakness (lack of transparency, political pressure etc.).
Evans (2020)	Guinea	Journal article	Unpacks WHO guidance for managing ethical issues in infectious disease outbreaks, specifically the issue on clinical data or sample sharing. Research cannot take place as challenges related to cold chain storage and samples safety is an issue in middle to low income countries, with insufficient infrastructure to carry out this kind of research.
Cardoen (2017)	Africa/Global	Journal article	Outbreak response should include surveillance, biosecurity measures, communication channels and training for personnel. Infrastructure be in place for safe, reliable and secure testing and stockpiling of pathogens.
Folayan (2018)	West Africa	Journal article	Research priorities were obtained from engaging experts and the community. Priorities need to focus on research which seeks to reduce morbidity and mortality in real-time. This includes prioritizing clinical trials that can help present day and not in future, which would aid in best utilizing limited resources. During the acute stage of outbreak, research must focus on patient care and outbreak control.
Nkengasong (2017)	Africa	Commentary	Speaks to strengthening health systems, but in terms of research, data-sharing must take place to prevent duplication.
African CDC (2017)	Africa	Scientific symposium report	Any public health research agenda has to be embedded in the 5 strategic pillars such as Surveillance and disease intelligence; Information system; Public health emergency preparedness and response; laboratory system and network; and Public health research and Institutes.
African Union (2015)	Africa	Strategic plan (Agenda 2063)	Investment in research infrastructure and support.
African Union (2016)	Africa	Policy framework (Africa Health 2016-2030)	Policy framework which includes supporting research and development of vaccines, medicines and technologies to address both communicable and non-communicable diseases.
WHO (2016)	Global (drawn from Africa)	Framework for general epidemic prevention.	Research to have improved coordination and foster global capacity to conduct research in the context of epidemics; implement research in a safe, effective, and timely manner; to develop and support research efforts and intervention.
Wellcome trust (2019)	Globa/Africa	Report	Highlights gaps of R&D, namely R&D as well as outbreak response to be contextualized to settings intervention needs to occur; there needs to be a focus on developing rapid diagnostic tests and point of care diagnostics.

**Table 3:** summary of evidence for included studies addressing zoonotic epidemic research agendas

Author (year)	Region of focus	Type of document	Key message
Kobres (2019)	Global (includes Africa)	Journal article	When reviewing research during the Zika outbreak, improvements suggested includes sharing data and code, developing standards, being able to post preprints to combat journal waiting time, but also communicating uncertainty when using prediction models.
Rojek (2017)	West Africa	Journal article	Need research on potential outbreaks linked to different species of Ebola; having comparable datasets available to compare key clinical findings; research must be protocol-directed, hypothesis driven.
Jacobsen (2016)	West Africa	Journal article	Research needs entail enhancing wildlife biosurveilance; expand on studies on ecological health interventions, improve predictive modelling; improve health systems and public health infrastructure; improve risk communication and the use of social media; the application of laboratory science; and improved diagnostic, therapeutic and preventative tools.
Abrahowitz (2018)	West Africa	Journal Article	Create systematic quantification of locally appropriate sociocultural factors; create interdisciplinary collaborations to refine “risk segmentation” methodologies and practices for real-world accuracy; develop qualitative indicators and composite social indexes to be used during epidemic outbreaks; Use untapped community resources to create real-time, data collection and response integration; create novel techniques for social mobilization and community engagement modelling; develop accurate, high-quality data collection and rapid development of multiple modeling frameworks as part of early emergency response; learn from the experience of the West Africa Ebola out-break of 2014-15.
Calnan (2018)	Guinea (West Africa)	Journal article	Developed seven research priority questions around the Ebola virus with key stakeholders, most pressing of which was the long-term socio-cultural, economic and health impact of the EVD epidemic on the country.
Wilder-Smith (2017)	West Africa	Journal article	Suggests critical assessment of vector control tools and those under development should guide a research agenda for determining which existing techniques work best, and how to best combine state-of-the-art vector control with vaccination.
Farrar (2015)	Global	Framework for Dengue	Research agenda aimed to optimize clinical management (research on patient care, diagnostics, standardized screening, ways to reduce mortality); evaluate staff training; better understand dengue pathogenesis; transmission research to focus on vector tools and surveillance; primary and secondary prevention to focus on vaccines and therapeutics development; health policy research to help redress priority of dengue, especially in less studied regions like Africa.
WHO Ebola Scientific Committee	West Africa/Global	Framework for Ebola	Seven research priorities identified on viral genetic diversity, biomarkers, public health practice, data, social and community interactions, promoting robust future research, looking at implementation science.
WHO (2016)	Global	Framework for Zika	Research to help understand virus characteristics and vector tools; to develop diagnostics, vaccines and therapeutics.

**Table 4:** summary of evidence for included studies addressing other specific epidemic research agendas

Author (year)	Region of focus	Type of document	Key message
Sylla (2012)	Africa	Journal article	Aligns message in part to findings from Gotch (2007) [19], that research must be linked to population-based information research. Research agendas should focus on prevention, maintain prominent focus on cancer etiology, and look at how known preventions can be implemented in an African context.
Chapman (2014)	Global/low-middle countries	Journal article	Established a list of 62 prioritized research questions based on 5 focal areas (abortion and unplanned pregnancy; Diabetes and other causes; labor and cesarean; postpartum hemorrhage and hypertensive disorders; health policy and system) to decrease maternal mortality rates.
Odone (2016)	WHO African region	Journal article	Study confirms that global priority research agenda set for HIV-associated TB, aligned to research needs of high TB burden countries. Namely, 60% of research priority areas such as TB prevention, intensified TB case finding, TB treatment for people living with HIV, drug-resistant TB and HIV, childhood TB and HIV, and integrated TB and HIV services had been conducted in the AFRO region, of which 70% were conducted in high TB burden countries.
Gouglas (2019)	Africa	Journal article	It would take an average $2.8-3.7 billion to support vaccine development for emerging infectious diseases prioritized by WHO, through to phase 2 trials. As such, through collaboration and guidance from regulators, this can streamline technical and regulatory pathways for feasible vaccine development. Furthermore, CEPI added 2 or so watch list diseases to priority infections in relation to recent outbreaks.
Oliveras (2018)	Southern Africa/Africa	Journal article	Propagates 6 elements essential for research participation and in clinical trial/research prioritization in HIV Youth research, namely participation needs to be Resourced, Impactful, Genuine, Harmless, Teen friendly, and Skills building (RIGHTS).
Krubiner (2019)	Africa/Global	Journal article	Presents 22 recommendations which advocate for ethical inclusion of the interests of pregnant women in the development and deployment of vaccines for emerging pathogens.
Papa (2015)	Africa	Journal article	Provides a 10year research roadmap purporting that Crimean-Congo hemorrhagic fever needs to focus on the vector-virus interaction, the impact of livestock and other animal infection, as well as vaccine development and treatment options.
Imrie (2013)	South Africa	Journal article	Ensure 'men-who-have-sex-with-men' are included into the HIV research agenda in order to ensure adequate response in sexual health, HIV prevention, treatment and care, public health services, and receive support.
Greenwood (2013)	Africa	Conference report	Identified research areas which includes good surveillance for meningitis; identification and validation of serological correlates of protection against meningococcal disease and carriage; the need for improved methods for detecting carriage; and determining the reasons underlying the epidemiology of meningococcal disease in the 'African meningitis belt'.
Yach (2014)	Global/South Africa	Journal article	Research to continue around tobacco control, but to assert more focus on trade agreements, prevention among women and girls, and to adequately address the impact of use (including long-term use) of new nicotine-delivery systems such as nicotine-based non-combustibles (including e-cigarettes).
Critchley (2017)	Sub-Saharan Africa	Journal article	For the research to determine the importance of transient hyperglycemia in patients with TB and Type 2 diabetes mellitus (DM) for global epidemic on multidrug resistant (MDR)-TB; clinical trial and large cohorts studies to focus on benefits of improved DM care and intensified TB treatment on TB/DM outcomes and the cost of screening methods for DM among new TB patients; the health impact and cost effectiveness in developing different interventions for routine TB-DM testing, treatment management, and treatment access.
Geng (2019)	Africa	Journal article	Research agenda to focus on the efficacy, efficiency, and the quality of HIV intervention implementation; be adaptive in strategies to cater to context to address diverse needs and minimize cost; for research to elevate the patient experience and to incorporate patient reported outcomes.

Surveillance was discussed in four sources, which focused more on biosurveilance of animal or livestock infections or monitoring a disease in the disease hotspot (e.g. meningitis in the African meningitis belt) [26,30,38,48]. Four sources spoke to services, namely in terms of aligning services to relevant populations into studies, integrating services, and optimizing clinical management and public health practice [22,33,49,50]. Three studies stressed the importance in creating standards, either through data collection, protocol eligibility, sharing data, score cards for comparative studies, or robust research [21,32,50]. Vaccine development was mentioned in relation to health policies in that it could help influence which vaccines get prioritised, but also to have pathways to vaccine development streamlined for timeous response. Other vaccine development factors include ethical inclusion of pregnant women into studies and the validation of serological correlates [23,29,30,38]. Furthermore, improved diagnostics and therapeutics are needed for better preparedness especially more rapid, point of care diagnostics are needed [26,51,52]. Two studies addressed transparent reporting in the use of predictive modelling, by improving the models and clearly communicating its finding including uncertainty [21,26]. Streamlined processes had been emphasized by two studies specifically, in order to efficiently begin vaccine development, resolve barriers, create case definitions and guidelines, pre-approved protocols, create mechanisms of exchange for data sharing [23,24].

Two studies mentioned the importance in research looking at the long-term, socio-cultural and health impacts these diseases have on the community or country but also measuring the impact on certain populations (e.g. tobacco research focusing on girls than boys due to increased prevalence) [21,40]. Research participation also received mention but mainly in terms of the ethical inclusion of vulnerable groups such youth participation in HIV research or pregnant women in vaccine research [28,29]. Only one study focused specifically on research priorities reducing maternal mortality [20]. Case management had been touched on by two documents linking to case finding, patient care, and screening [22,49]. Lastly, vector control was mostly discussed in terms of Ebola and Zika, and it being combined with vaccine development for improved response [35,51].

## Discussion

This scoping review identified 34 documents deemed to be eligible for inclusion. Majority of the research agendas were derived from documents dealing with general epidemic or pandemic response [24,32,34,36,37,39,41-44,47,52], followed by research agendas drawing from lessons learned from the Ebola outbreak [25,27,31,35,50] which greatly informed response to the Zika outbreak which followed soon after [21,51]. General epidemic preparedness is needed as emerging and re-emerging diseases are increasing especially in developing countries [53]. This general framework equips health systems to have general preparedness as described by Fatiregun and colleagues [54] whereby epidemic management guides all activities from the national to health facility level, to adequately detect disease outbreaks, effectively respond to it by ensuring staff are well organized to investigate and thus effectively respond to disease outbreak.

Of the included studies, the broad manner in which transmission was addressed throughout the documents, illustrates the various levels at which one should understand the characteristics of the pathogen, and mechanisms of transmission in successful isolation and limiting of the disease spread [4]. Furthermore, understanding transmission falls part of early detection of which a new disease might mean new interventions [4]. This in turn links to infrastructure in building research capacity, which too was largely addressed by most of the documents, which would allow for mobilization and rapid deployment of and testing of candidate products within countries where outbreaks are most likely to occur [55]. An example of this lack of infrastructure goes back to the Ebola outbreaks, which delayed response in the form of vaccine and therapeutics ultimately resulting in a hindered outcome impact [55].

In having research agendas, we allow opportunities to bridge medium- and long-term knowledge gaps [56]. Yet in respect to epidemic research, research agendas create a harmonized response with key stakeholders. Epidemic research agendas allow high-quality research protocols and tools to be shared whilst inviting materials and samples from research partners in an ethical manner [56]. However, Africa needs to create its own health research agendas and capacitate itself to conduct and lead these studies, as the notion of 'safari science' whereby African scientists act as data gatherers for Western research agendas, is not an effective route any longer [57]. African health research decisions must center on Africa, with African researchers taking the lead not only on the science produced but ensuring inclusive and equitable involvement from fellow researchers, and in engaging national health ministries as well as the communities [57]. This engagement with community needs and contextualized interventions had been highlighted in four documents included in this study [18,26,46,52]. These may in turn further build sustainability and local commitment, which must transcend the need of funders or any project [57].

**Limitations:** this review, though attempting a highly sensitive search to capture as much eligible evidence as possible, may not have located all sources, especially when searching grey literature, in which some documents may be missed.

## Conclusion

It is vital for Africa to take the lead on its health decisions as well as lead the science in Africa's epidemic response. However, to do this we must ensure we are capacitated to do so and have the infrastructure for secure and adequate response and mobilization of real-time research and deployment of vaccines or other therapeutic products.

### What is known about this topic

- Research agendas are vital for a coordinated epidemic response;
- Low to middle income countries are limited in ability to carry out necessary outbreak surveillance and research activities.

### What this study adds

- An overview of available research agendas focusing on African relevant outbreaks and opportunities for research preparedness.
- This study highlights the need for context relevant outbreak research agendas and the need for sufficient infrastructure to address them.
